# Improving sleep health through sleep hygiene education in adults aged 50–80 years

**DOI:** 10.3389/frsle.2025.1722557

**Published:** 2026-01-14

**Authors:** Ashley M. Pfeiffer, Craig Triplett, Olivia Schaefers

**Affiliations:** Department of Exercise Science, School of Behavioral Sciences, Black Hills State University, Spearfish, SD, United States

**Keywords:** behavioral intervention, older adults, sleep education, sleep hygiene, sleep quality

## Abstract

**Introduction:**

Sleep plays a critical role in maintaining physical and cognitive health in older adults, yet sleep problems are highly prevalent in this population. Conventional management strategies often rely on pharmacological interventions, which may cause adverse side effects, evidencing the need for safe, low-cost alternatives. Sleep hygiene education offers a promising approach, and this study evaluates the efficacy of a strategy combining a one-time educational video and daily automated text messages in improving sleep quality, daytime sleepiness, and overall sleep hygiene practices among older adults.

**Methods:**

Participants completed an electronic survey that collected demographic information and included the Pittsburgh Sleep Quality Index (PSQI), Epworth Sleepiness Scale (ESS), Sleep Hygiene Index (SHI), Perceived Stress Scale, and Numeric Pain Rating Scale. Each participant wore a Fitbit sleep tracker for 2 weeks to establish baseline data on total sleep time, time awake, time in rapid eye movement (REM)/light/deep sleep, and sleep efficiency. Participants were then randomly assigned to a control group, a video-only group, or a video-plus-text group. Sleep tracking continued for 4 additional weeks, and all assessments were repeated at the end of the study.

**Results:**

A total of 119 participants (mean age 66.5 ± 7.2 years; 77 females) completed the study. Paired *t*-tests compared pre- and post-intervention scores. Both the video-only and video-plus-text groups showed significant improvements on the PSQI, ESS, and SHI compared to the control. No significant changes were found in measured objective sleep parameters.

**Discussion:**

Findings indicate that video-based sleep hygiene education, with or without supplemental text messaging, was associated with improvements in subjective sleep quality, daytime sleepiness, and sleep hygiene behaviors. However, these improvements were not reflected in objective sleep measures, highlighting a discrepancy commonly reported in sleep research.

**Conclusion:**

Brief, low-cost sleep hygiene education interventions, delivered through video or a combination of video and text messaging, may improve perceived sleep quality and sleep-related behaviors in older adults.

## 1 Introduction

Sleep is critically important for the physical and mental health of adults aged over 50 (Corbo et al., 2023; Jaqua et al., 2023; Mander, 2020; Tian et al., 2024). Poor sleep has been linked to depression, anxiety, dementia, obesity, hypertension, cardiovascular disease, stroke, falls, and reduced quality of life (Chaput et al., 2023; Dzierzewski et al., 2022; Huang et al., 2022; Knechel and Chang, 2022; Zhang et al., 2022). Adequate sleep also plays a vital role in attention, cognition, learning, memory, and recovery (Chen et al., 2024; Dahat et al., 2023; Hyndych et al., 2025; Al-Sharman and Siengsukon, 2013; Venneman, 2023; Zimmerman et al., 2024).

Sleep problems are highly prevalent among older adults: up to 50% report difficulty falling or staying asleep, 40% report poor sleep quality, and 30% experience excessive daytime sleepiness (Hinz et al., 2017; Jaqua et al., 2023; Jaussent et al., 2017). As Covassin and Singh (2016) emphasized, “Given the pervasive and escalating prevalence of inadequate sleep… the potential future burden on public health cannot be ignored.” Despite this prevalence, sleep issues in older adults are often overlooked in routine care. When addressed, they are commonly treated with pharmacological interventions, which may cause adverse side effects (Espeso and Wick, 2023; Gordon et al., 2022; Heun-Johnson et al., 2025).

There is a clear need for alternative interventions to improve the quality of sleep in older adults and to mitigate the broader consequences of poor sleep on their health and daily activities. Education is particularly valuable, as it can help older adults recognize that sleep health is a modifiable health behavior and effective alternative solutions such as sleep hygiene education may be appropriate intervention strategies. Sleep hygiene generally refers to behavioral and environmental practices that support healthy sleep, including maintaining a regular sleep schedule, developing a relaxing bedtime routine, and optimizing the sleep environment. Although widely used, the term, Sleep hygiene, lacks a universally accepted definition, and the specific practices included can vary (De Pasquale et al., 2022).

Murawski et al. (2018) reviewed interventions aimed at improving sleep health in adults and found that stress management and relaxation, stimulus control, exercise, and sleep hygiene were the most common strategies. Within older adults, recent studies have demonstrated that sleep hygiene education delivered either through video-based formats or one-time in-person sessions can improve sleep hygiene behaviors and sleep quality (Carvalho et al., 2022; Tucker et al., 2021). Another behavioral change strategy that has not yet been explored in older adults is automated messaging, which has shown promise in improving sleep hygiene and sleep quality in college-aged students (Bani Issa et al., 2023).

The primary aim of this randomized controlled trial was to evaluate the efficacy of sleep hygiene education in adults aged 50–80. The intervention included sleep hygiene education in video and text message formats, along with sleep tracking using subjective questionnaires and objective data from Fitbit sleep trackers. The study hypothesized that participants receiving these interventions would demonstrate significant improvements in sleep quality, daytime sleepiness, and adherence to sleep hygiene practices, compared with the control group. Furthermore, we anticipated that the intervention groups would show statistically significant improvements across both subjective and objective measures of sleep health.

## 2 Materials and methods

### 2.1 Study design

This study used a randomized controlled trial design with two experimental groups and one control group. Both objective and subjective measures were used to assess participants' sleep health, including sleep quality, self-reported habits, and perceived sleep experiences. Data were collected over a 6 week period. In an a priori power analysis for a two-group comparison, the significance level was set at α = 0.05 (two-tailed), and a medium effect size of d = 0.50 was assumed, resulting in a required sample of 48 participants per group and a total sample size of 144 participants, which yielded an estimated statistical power of 92.4%. Simple randomization was used. A computer-generated random number sequence with a 1:1 allocation ratio was created by an investigator not involved in participant enrollment. Allocation was also generated by an investigator not involved in participant enrollment or data collection. The study coordinator enrolled participants, and assignments were revealed after enrollment.

### 2.2 Participants

Community-dwelling adults aged 50–80 were recruited from the Midwestern United States. Recruitment strategies included posters, social media posts, and word-of-mouth referrals. Eligibility criteria required participants to be between the ages of 50–80, living independently, and have access to a smartphone. Individuals with an untreated, clinically diagnosed sleep disorder were excluded.

Eligible participants completed an initial meeting with the research team and an online survey consisting of standardized sleep questionnaires. Surveys were administered through PsychData (PsychData.com, 2025), a secure, web-based data collection system.

The study protocol was approved by the Institutional Review Board at Black Hills State University in Spearfish, South Dakota. The authors declare that the research was conducted in the absence of any commercial or financial relationships that could be construed as a potential conflict of interest. All study data is available from the authors upon request.

### 2.3 Questionnaires

The survey consisted of the following components: (1) demographic information, (2) a medical history questionnaire, (3) a medication questionnaire, (4) behavior-based questions assessing readiness to change, and (5) validated instruments measuring sleep quality, sleep habits, daytime sleepiness, and perceived stress levels.

Participants were asked to provide demographic information, including age, sex, ethnicity, race, highest level of education completed, employment status, marital status, and average pain severity over the past month, measured with the Numeric Pain Rating Scale (Jensen et al., 1986). The medical history questionnaire assessed comorbidities such as depression, anxiety, fibromyalgia, chronic pain, lung disease, arthritis, cancer, diabetes, kidney disease, neurological conditions, and thyroid disorders.

The sleep-related questionnaires in the survey included the Pittsburgh Sleep Quality Index (PSQI) (Buysse et al., 1989), Epworth Sleepiness Scale (ESS) (Johns, 1992), Sleep Hygiene Index (SHI) (Mastin et al., 2006), and Perceived Stress Scale (PSS) (Lee, 2012).

The PSQI is a 24-item survey that measures sleep disturbances across seven components: subjective sleep quality, sleep latency, sleep duration, habitual sleep efficiency, sleep disturbances, use of sleep medication, and daytime dysfunction. These seven areas are summed to produce an overall score. Responses are based on the majority of days (and nights) over the previous month (Buysse et al., 1989). The PSQI includes 19 questions, with overall scores ranging from 0 to 21; higher scores indicate poorer sleep quality. The PSQI has demonstrated good reliability and validity across diverse populations (Backhaus et al., 2002; Buysse et al., 1989; Carpenter and Andrykowski, 1998; Fictenberg et al., 2001; Gentili et al., 1995; Grandner et al., 2006).

The ESS assesses daytime sleepiness in adults (Johns, 1992). It comprises eight questions, with scores ranging from 0 to 24; higher scores reflect higher levels of daytime sleepiness (Johns, 1992). The ESS has also shown good reliability and validity across diverse populations (Beiske et al., 2009; Izci et al., 2008; Johns, 1992; Manzar et al., 2019; Rosca et al., 2011).

The SHI is a 13-item self-report measure of participants' sleep hygiene behaviors. Scores range from 0 to 52, with higher scores reflecting poorer sleep hygiene (Seun-Fadipe et al., 2018). The SHI has also demonstrated good reliability and validity across diverse populations (Cho et al., 2013; Mastin et al., 2006; Meltzer et al., 2014; Seun-Fadipe et al., 2018).

The PSS assesses how the perception of a situation can influence self-reported stress levels, with classification into low, moderate, or high stress categories. Participants were asked about their feelings and thoughts during the past month. Each item was rated on a five-point scale from 0 (never) to 4 (very often). Total scores range from 0 to 40, with 0–13 indicating low perceived stress, 14–26 moderate perceived stress, and 27–40 high perceived stress (Nielsen et al., 2016).

Additionally, at the end of the post-test survey, all participants were asked, “Please rate your ability to change any of your sleep behaviors throughout this study.” Responses were ranked on a scale of 0 to 10, with 0 being no confidence in their perceived ability to change their sleep behaviors and 10 indicating extremely high confidence. The importance of this question can be demonstrated by recent research showing that the perceptions of sleep play a critical role in wellbeing. This research also suggests that future studies should aim to improve both subjective sleep outcomes and perception of sleep (Lenneis et al., 2024).

The intervention in this study consisted of two possible delivery modes to educate participants on the importance of sleep hygiene behaviors. The educational tips were drawn from the works of leading researchers in the field of sleep hygiene (Barnes and Drake, 2015; Boubekri et al., 2020; Siengsukon et al., 2017). The first mode of delivery was an eight-minute video, which presented 15 sleep hygiene tips, such as “Go to sleep and wake up at the same time every day. This will help set your natural biological clock” (Table 1). The second intervention group also watched the same educational video; however, their learning was supplemented with daily automated text messages that reinforced the video content. These messages were sent every evening at 8:00 p.m. local time for 4 weeks via the automated software platform, Fitabase.

**Table 1:** List of sleep hygiene educational recommendations.

**Go to sleep and wake up at the same time every day. This will help set your natural biological clock**.
Exposure to bright natural light when you first wake up is also helpful to set your natural biological clock.
Use your bed for only sleep and sexual activity to help train your brain that if you are in your bed, you should be sleeping. Do not eat, work, or watch TV in bed. Do these activities outside of the bedroom.
Leave bed if unable to fall asleep within 20 mins and return when sleepy. If unable to leave the bed due to limited mobility or safety concerns, do something relaxing (i.e., relaxation techniques) until sleepy and able to fall to sleep.
Develop a relaxing bedtime routine. This may include taking a warm bath, reading a book, meditation, or stretching. Avoid stimulating activities right before bedtime, including watching TV or discussing a stressful topic.
Avoid moderate to vigorous exercise at least 2-3 h before bedtime. Exercising immediately before bedtime stimulates your body and brain, making it hard to fall asleep. There is evidence however that doing regular (preferably moderate to vigorous) exercise improves your sleep at night. Talk to your physical therapist about an appropriate exercise program.
Avoid caffeinated foods and drinks at least 4 h before bedtime (includes most tea, coffee, chocolate, and soft drinks). Check the presence of caffeine in your drink or food by reading the label. Caffeine can cause difficulty falling asleep and increase the number of times you wake up during the night.
Refrain from drinking alcohol or smoking at least 3 to 4 h before bedtime. Although people may think drinking alcohol causes relaxation before bedtime it can actually increase the number of times you wake up during the night and can cause you to wake up early. Nicotine in cigarettes acts as a stimulant that can cause difficulty falling asleep.
Do not take unprescribed or over-the-counter sleeping pills.
Avoid daytime napping so that you are tired at night and can fall asleep easily. If you feel you need to take a nap, limit the nap to 30 mins and avoid napping in the evening.
Make your sleeping environment comfortable and relaxing. This includes avoiding too much light and disturbing noises. Stop using light emitting electronics (i.e., television, computer, smartphone) at least 30 mins before bedtime as the blue light that is emitted can disrupt sleep by suppressing melatonin production. Use ear plugs, light-blocking curtains, or an eye mask if needed.
Keep the temperature comfortable. Being too warm or cold may disturb your sleep. Also, use a comfortable and supportive pillow and mattress.
Avoid eating a large meal or spicy food 2-3 h before going to bed. Your digestive system slows down while you are sleeping, which can stimulate acid secretions that cause heartburn. A light snack may be helpful if you are hungry. Avoid excessive liquid 2-3 h before bedtime.
Eat nutritious, well-balanced meals. Avoid eating a large amount of highly processed and sugary foods as these can disrupt the hormones that help regulate your sleep patterns. Instead try to make a habit of eating whole foods whenever possible.
Talk to your doctor or health professional if you still have trouble sleeping.

After completing the survey, participants were randomly assigned to one of three groups. All participants were provided with a sleep tracker. Among the various consumer wearable devices that track sleep, Fitbit is used in more biomedical clinical research than any other consumer wearable (Henriksen et al., 2018). The Fitbit Inspire HR 2 was specifically selected for its demonstrated ability to track sleep habits and because it most closely aligns with the now-discontinued Fitbit Alta, which was found to be comparable—and slightly more accurate—to Actigraphy (Gorzelitz et al., 2020; Chinoy et al., 2021).

All participants were instructed to wear their Fitbit continuously for 6 weeks. During the first 2 weeks, the devices recorded baseline sleep data. After this baseline period, participants in the two intervention groups received their respective interventions. At the conclusion of the 6 week study, participants were also asked these two additional questions: (1) “Please rate your ability to change any of your sleep behaviors throughout this study,” and (2) “Please rate your satisfaction with the sleep hygiene video and text messages.” Both questions were rated on a 0–10 scale, with higher scores indicating greater perceived ability to change and greater satisfaction.

### 2.4 Data analysis

Survey data were downloaded from PsychData and analyzed using IBM SPSS Statistics version 28 (SPSS Inc., Cary, NC). Only participants who completed the entire study were included in the analyses. Descriptive statistics were used to describe participant characteristics. Means and standard deviations (SDs) were calculated for continuous variables, and frequency distributions were calculated for categorical data. Differences between pre- and post-intervention were tested for both subjective and objective outcomes using paired-sample *t*-tests. *P*-values < 0.05 were considered statistically significant.

## 3 Results

### 3.1 Participant demographics

**Figure 1:**
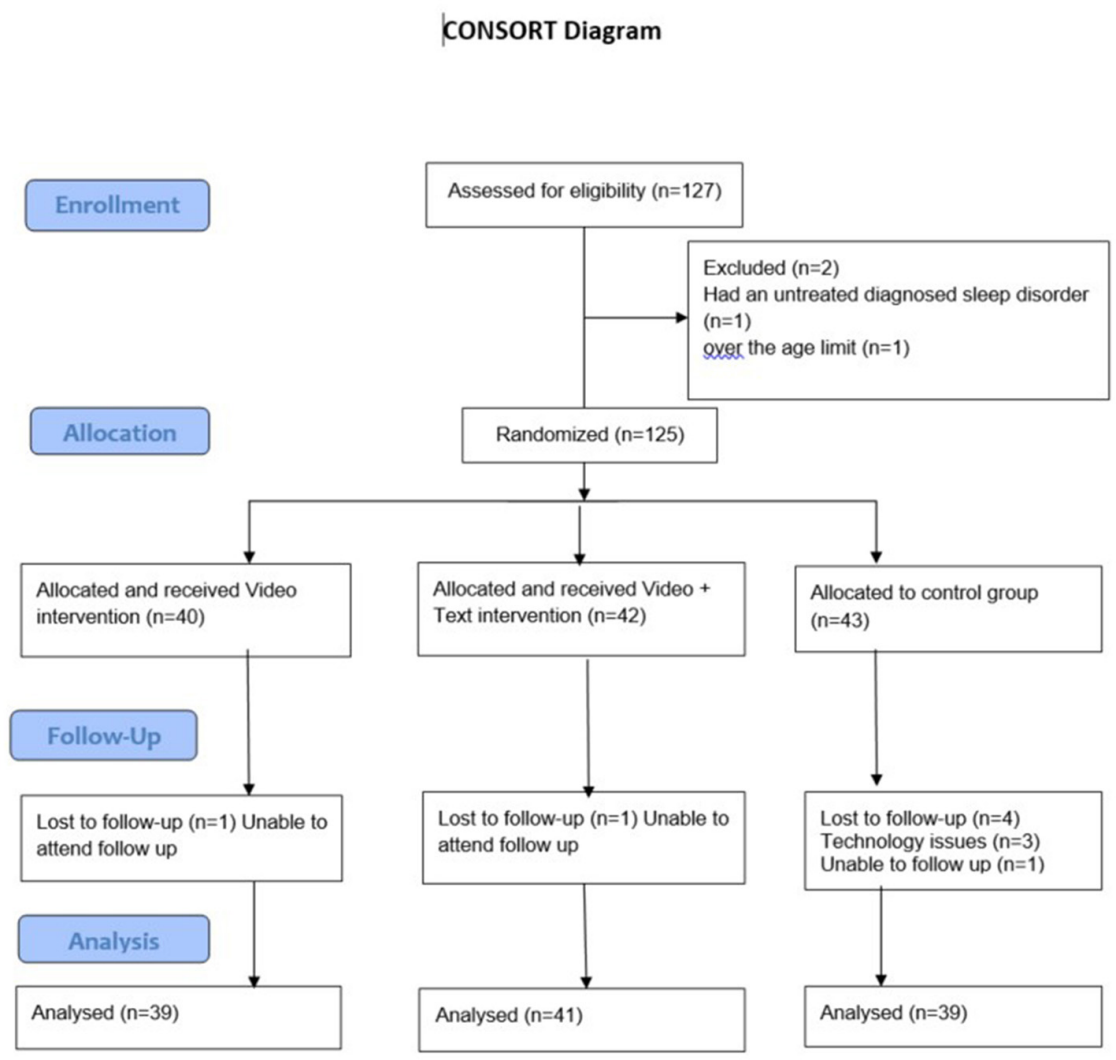
CONSORT diagram.

A total of 125 participants were recruited and enrolled in the trial; 119 completed the study. Six participants dropped out of the study prior to follow up data collection, three of which were unable to attend a follow up and three due to technology issues. As a result 41 out of 42 participants completed follow up testing for the text message group, 39 out of 40 for the video-only group, and 39 out of 43 for the control group (Figure 1). Due to slower-than-anticipated recruitment, the final sample did not reach this target, which may have reduced the statistical power to detect small-to-moderate effects.

Demographic characteristics are presented in Table 2. Among the 119 participants who completed the study, 77 (64.7%) identified as female. The mean age was 66.5 years (SD = 7.20), with ages ranging from 50 to 79 years. The majority of participants were White (*n* = 115, 96.6%), had a college degree (*n* = 88; 74%), were retired (*n* = 67; 56.3%), and were married (*n* = 89; 74.8%). Comorbidity data were collected for all participants. The conditions assessed included: diabetes, depression, cancer, arthritis, neurological disease, thyroid disease, hypertension, heart disease, fibromyalgia, and chronic pain. Of the total sample, only 20 participants reported no comorbidities; 3 of those were in the video group, 10 were in the text-messaging group, and 7 were in the control group. When comparing the prevalence of comorbidities in each group they were fairly equally distributed. For example, 29 reported experiencing symptoms of depression. Among those reporting depression, 9 were in the control group, 10 were in the video group, and 10 were in the text-messaging group. Because the prevalence of depression was comparable across the three groups, additional between-group analyses were not conducted. Moreover, given that only 20 participants reported having no comorbidities, further subgroup analyses based on comorbidity-free status were not pursued (Table 2).

**Table 2:** Demographic characteristics of the study population (*N* = 119).

**Variable**		***n***	**%**
Age (mean 66.5 ± 7.2 years)			
Gender	Male	41	34.5
	Female	77	64.7
	Other	1	0.8
Ethnicity	Hispanic or latino	1	0.8
	Not hispanic or latino	118	99.2
Educational level	GED or high school	16	13.4
	Some college classes, no degree	12	10.1
	Associate degree	16	13.4
	Bachelor's degree	39	32.8
	Master's degree	20	16.8
	Doctoral degree	13	10.9
	Other	3	2.5
Employment status	Retired	67	56.3
	Not working	1	0.8
	Full time employee	18	15.1
	Part time employee	31	26.1
	Other	2	1.7
Marital status	Single	9	7.6
	Married	89	74.8
	Divorced, separated	12	10.1
	Not married, widowed	9	7.6
Comorbidities	Depression	29	24.4
	Fibromyalgia	3	2.5
	Chronic pain	31	26.1
	Lung disease	18	15.1
	Arthritis	57	47.9
	Cancer	33	27.7
	Cardiovascular disease	42	35.3
	Diabetes	6	5.0
	Kidney Disease	1	0.8
	Neurological Disease	10	8.4

### 3.2 Subjective sleep measures

Both intention-to-treat and per-protocol analyses were conducted for all subjective sleep outcome measures.

#### 3.2.1 PSQI

Both intervention groups demonstrated statistically significant improvements in sleep quality as measured by the PSQI. The video-only group showed a reduction in PSQI scores from 6.82 to 5.82 (*p* = 0.048), indicating improved sleep quality. Similarly, the video-plus-text-message group showed a reduction from 7.63 to 6.51 (*p* = 0.017). In contrast, the control group showed a non-significant increase in PSQI scores from 7.23 to 7.84 (*p* = 0.211), suggesting a slight worsening of sleep quality.

#### 3.2.2 SHI

Both intervention groups demonstrated significant improvements in sleep hygiene practices. On the SHI, lower scores indicate better sleep hygiene practices. The video-only group's scores decreased from 11.38 to 8.97 (*p* = 0.013), and the video-plus-text-message group's scores decreased from 12.44 to 10.46 (*p* = 0.022). The control group showed a non-significant improvement, with scores decreasing from 11.46 to 10.82 (*p* = 0.296).

#### 3.2.3 ESS

Daytime sleepiness, as measured by the ESS, significantly improved in both intervention groups. The video-only group's ESS scores decreased from 6.97 to 5.36 (*p* = 0.004), and the video-plus-text-message group's scores decreased from 6.98 to 5.27 (*p* < 0.001), indicating reduced daytime sleepiness. The control group again showed a non-significant improvement, decreasing from 6.62 to 6.23 (*p* = 0.495).

#### 3.2.4 PSS

Interestingly, only the video-plus-text-message group showed a significant reduction in perceived stress, with PSS scores decreasing from 20.95 to 19.24 (*p* = 0.02). The video-only group showed a slight, non-significant increase from 20.87 to 20.97 (*p* = 0.867), while the control group showed a non-significant decrease from 20.15 to 19.92 (*p* = 0.652).

#### 3.2.5 Behavioral change perception

Both intervention groups showed higher self-perceived ability to change sleep behaviors compared with the control group. On the 10-point Likert scale (0 = no confidence; 10 = extremely confident), the video group had an average score of 4.3, and the video plus text message group an average of 5.2. In contrast, the control group had a lower average score of 2.2.

#### 3.2.6 Intention-to-treat analyses

Intention-to-treat analyses were also run and showed similar findings to the per-protocol analyses. The video-only group showed a reduction in PSQI scores from 7.10 to 6.20 (*p* = 0.03), while the video-plus-text-message group showed a reduction from 7.60 to 6.52 (*p* = 0.01). In contrast, the control group scores increased from 7.05 to 7.37 (*p* = 0.24). For the SHI, the video-only group's scores decreased from 10.80 to 8.90 (*p* = 0.01), the video-plus-text-message group's scores decreased from 12.48 to 10.52 (*p* = 0.01), and the control group went from 11.07 to 9.90 (*p* = 0.053). For the ESS, the video-only group decreased from 6.73 to 5.40 (*p* = 0.002), the video-plus-text-message group decreased from 7.05 to 5.29 (*p* < 0.001), and the control group decreased from 6.73 to 6.51 (*p* = 0.36). Lastly, the PSS video-only group's scores increased from 20.51 to 20.64 (*p* = 0.42), and the control group increased from 20.39 to 20.12 (*p* = 0.289). Only the video-plus-text-message group decreased their PSS scores from 21.12 to 19.24 (*p* = 0.006).

### 3.3 Objective sleep measures

Despite the significant improvements observed in subjective sleep measures, none of the groups showed statistically significant changes in objective sleep parameters as measured by the Fitbit sleep tracker. Intention-to-treat analyses could not be performed on objective measures due to participants not staying in the study long enough for sleep tracker to track this data.

#### 3.3.1 Total sleep time

The video-only group showed a slight increase in total sleep time from 402.63 to 405.59 mins (*p* = 0.728). The video-plus-text-message group showed a decrease from 414.65 to 406.40 mins (*p* = 0.365), while the control group showed an increase from 398.33 to 402.63 mins (*p* = 0.562).

#### 3.3.2 Sleep architecture

No significant changes were observed in time spent in deep sleep, light sleep, or rapid eye movement (REM) sleep across any of the groups. The video-only group showed minimal changes in deep sleep (56.16 to 57.68 mins, *p* = 0.519), light sleep (267.47 to 272.66 mins, *p* = 0.460), and REM sleep (75.54 to 75.85 mins, *p* = 0.920). Similar non-significant changes were observed in both the video-plus-text-message group and the control group.

#### 3.3.3 Sleep efficiency

Sleep efficiency remained relatively stable across all groups. The sleep efficiency of the video-only group went from 87.17% to 87.19% (*p* = 0.966), as did the video-plus-text-message group, going from 86.98% to 86.93% (*p* = 0.869), and the control group, from 87.03% to 87.40% (*p* = 0.294).

#### 3.3.4 Total time awake

No significant changes were observed in total time awake during the night. The video-only group showed a slight increase from 62.12 to 64.98 mins (*p* = 0.438), while the video-plus-text-message group increased from 60.95 to 62.68 mins (*p* = 0.397). The control group decreased from 58.11 to 55.79 mins (*p* = 0.286).

## 4 Discussion

Previous studies have linked better subjective sleep quality to broader quality of life benefits, including improvements in mental health, life satisfaction, and overall subjective wellbeing. A 2021 meta-analysis suggested that healthcare professionals should strengthen their capacity to provide sleep education and implement interventions aimed at improving patients' sleep quality and overall sleep health (Scott et al., 2021). More recently, a 2025 study trialed sleep hygiene education in participants diagnosed with an insomnia sleep disorder and found significant improvements in sleep quality and daytime sleepiness (Hafizoglu et al., 2025). The current study examined the effectiveness of sleep hygiene education on sleep behaviors in older adults living independently in the community. Both intervention groups (video-only and video-plus-text-message) demonstrated statistically significant improvements in subjective sleep measures, while the control group showed no significant changes, when comparing pre- and post-test data. PSQI and SHI scores decreased in both intervention groups, indicating that participants perceived improvements in sleep hygiene behaviors and quality. The ESS demonstrated the most consistent improvement across the intervention groups, reflecting reduced daytime sleepiness. These findings align with previous research supporting the efficacy of educational interventions for improving subjective sleep quality in older adults (Corral-Pérez et al., 2024; Tucker et al., 2021).

Although paired-samples *t*-tests revealed positive results, these findings cannot be generalized to all participants in the intervention groups. It appears that these interventions were effective for some of the individuals, but not universally across all participants. Future research should investigate this further with the inclusion of larger sample sizes and long-term follow-up to determine the sustainability and generalizability of these improvements.

Interestingly, the PSS showed mixed results, with no significant change in the video-only group but a significant reduction in the video-plus-text-message group. Further testing is needed to explore whether the addition of text messages provides added benefits for stress management.

Despite improvements in subjective measures, objective sleep parameters measured by Fitbit showed no statistically significant changes in any group. Total sleep time, sleep efficiency, and time spent in different sleep stages remained relatively stable across all conditions. The discrepancy between subjective and objective measures suggests that, while participants perceived improvements in their sleep quality and related factors, these perceptions may not have been reflected in actual sleep architecture or efficiency as measured by the tracking device.

This discrepancy between subjective and objective sleep measures is consistent with prior sleep intervention research (Cudney et al., 2022; Trimmel et al., 2021). Lund et al. (2013) similarly reported improvements in perceived sleep quality without corresponding changes in sleep tracker-measured sleep parameters. This phenomenon may reflect changes in sleep perception and attitudes rather than fundamental alterations in sleep architecture (Kölling and Hof zum Berge, 2020).

## 5 Limitations

Several limitations should be considered when interpreting these results. First, the sample was predominantly female, well-educated, and non-Hispanic White, which limits the generalizability of findings to more diverse populations of older adults. Additionally, participants who volunteered may have been more motivated to improve their sleep than the general older adult population, introducing potential selection bias.

Second, outcomes were assessed only immediately following the intervention, without long-term follow-up, leaving questions about the durability of improvements.

Third, the discrepancy between subjective and objective outcomes raises questions about the mechanisms underlying perceived improvements. As is the case in many studies of this nature, a potential Hawthorne effect may also have influenced participants' subjective reporting, as awareness of being in a sleep-related study could have affected their responses.

Lastly, although demographic data were collected, not all health conditions that might influence sleep-related outcomes were accounted for, which could have introduced uncontrolled variability into the results.

## 6 Future directions

This study suggests several avenues for future research. First, a longer-term follow-up study is needed to evaluate the sustainability of the improvements observed in this shorter-duration intervention. Researchers could also explore the mechanisms underlying the subjective—objective discrepancy and develop strategies to translate these into measurable changes in objective sleep parameters.

This study could be expanded to include more diverse populations, particularly those with lower educational attainment, to improve generalizability. Examining tailored interventions based on specific sleep complaints is another potential direction. Further studies assessing dose—response relationships could help determine the optimal intervention duration.

Also, further investigation into the integration of sleep hygiene education with other behavioral interventions, such as stress management and physical activity, may help clarify whether combined approaches elicit more significant changes in sleep health.

## 7 Conclusion

This study demonstrates that brief video-based sleep hygiene education interventions may improve subjective sleep quality, sleep hygiene behaviors, and daytime sleepiness in older adults. However, further research is needed to confirm these preliminary findings. While objective sleep parameters remained unchanged, the improvements in perceived sleep quality and daytime functioning are promising and warrant continued investigation.

## Data Availability

The original contributions presented in the study are included in the article/supplementary material, further inquiries can be directed to the corresponding author.
